# It’s plain and simple: transparency is good for science and in the public interest

**DOI:** 10.1186/1745-6215-14-215

**Published:** 2013-07-12

**Authors:** Simon Denegri, Helene Faure

**Affiliations:** 1 Chair INVOLVE, NIHR National Director for Public Participation and Engagement in Research, Involve, 33 Corsham Street, London N1 6DR, United Kingdom; 2 Database Manager Current Controlled Trials, BioMed Central, 236 Gray's Inn Road, London WC1X 8HB, United Kingdom

**Keywords:** Trial, Transparency, Plain english, Lay summary, Guidance

## Abstract

In the past couple of years, there has been a growing focus on the need to make scientific output accessible to a greater number of people, especially in the field of clinical research. The public are being urged to become more well-informed and to ask their doctors about taking part in clinical trials.

A key finding of a report from the Association of Medical Research Charities was that all published scientific papers would benefit from having a section in plain English. Researchers running a clinical trial are expected to provide a summary of their intended research at various stages of the research process. However, there is evidence that existing summaries are of variable length and quality and not always in plain English.

As a result, the National Institute for Health Research (NIHR) commissioned a review of the guidance that is available to researchers. However, recent initiatives demonstrate that there are still a number of challenges in making current research both accessible and understandable by prospective participants.

BioMed Central also has a number of ongoing initiatives involving trial registration services and journals.

## Background

In the past couple of years, there has been a growing focus on the need to make scientific output accessible to a greater number of people, especially in the field of clinical research.

Under the NHS Constitution, patients have the right to be informed about relevant and appropriate clinical research [1]. The public are being urged to become more well-informed and to ask their doctors about taking part in clinical trials. The debate about access to research results has led to a House of Commons Select Committee inquiry [2]. This short article looks at a number of recent initiatives in the UK and explains how open-access publisher BioMed Central is approaching the task.

In 2011, the National Institute for Health Research (NIHR) launched a website listing all clinical trials that have recruited or are actively recruiting participants in the UK, the UK Clinical Trials Gateway website [3]. The information on this website comes from two publicly available sources of information, ClinicalTrials.gov [4] and the International Standard Randomised Controlled Trial Number Register (ISRCTN) [5], which were primarily set up to ensure that researchers were open about their research and to allow health professionals reviewing clinical evidence to bridge the gap between published and unpublished research. The registers have since evolved to become a point of reference for patients and the general public.

### Summaries in plain English

Also in 2011, the Association of Medical Research Charities [6], in collaboration with the British Library and the United Kingdom Office for Library and Information Networking (UKOLN) at the University of Bath, started the *Patients Participate!* initiative, which examined how patients and the general public could be given the right tools to make sense of the increasing number of research outcomes as reported in the press and media. One of the key findings of their report was that all published scientific papers would benefit from having a section in plain English, which is currently far from being the case.

Researchers running a clinical trial are expected to provide a summary of their intended research at various stages of the research process (funding grant application, regulatory process, ethics approval, and so on). These requirements are likely to differ from one stage to the next. In 2013, there is evidence that existing summaries will be of variable length and quality and not always in plain English. The UK Clinical Trials Gateway may meet its objective of providing a one-stop environment for trials recruiting in the UK but, by reusing the content found under the ‘lay summary’ or ‘research summary’ sections, as required by a number of research funders, it is highly unlikely to meet its objective of providing patients and the public with information in an accessible and clear format. Conversely, if the UK Clinical Trials Gateway really does aim to maximize participation in clinical research, this will raise the bar in terms of lay-friendliness of the information that is presented.

### Guidance for researchers

As a result, the National Institute for Health Research (NIHR) commissioned a review of the guidance that is available to its own researchers. INVOLVE, the NIHR-funded body that promotes the involvement of the public, consulted a number of stakeholders and in January 2013 produced a report [7] stressing the needs:

●  To explain to researchers why summaries in plain (rather than scientific) English are important.

●  To give advice on how to write in plain English [8].

●  To aim for content that uses English at the same level as would be found in a broadsheet newspaper.

Ensuring that future plain English summaries meet quality standards could become a requirement for NIHR funding, although the detail of implementation has yet to be determined.

### Journal involvement

The NIHR is also building on the reputation of the highly regarded journal *Health Technology Assessment*[9] and will very soon be launching its own library of journals [10]. This will ensure that all NIHR-funded research can be disseminated in a timely and permanently accessible manner. This implementation will rely on the input of both health professionals and members of the public.

As a publisher of over 160 journals in the field of medicine, BioMed Central is actively involved in improving clinical trial transparency via its open-access model [11]. BioMed Central administers the ISRCTN register. This is very much seen as the first step towards transparency and future dissemination of research outcomes. Each ISRCTN record has a dedicated field that can be used by researchers to describe their work in plain English, following guidelines adopted from the CancerHelp UK model [12].

### Perceived difficulties

Some journals within the BioMed Central portfolio already offer the option of providing a lay abstract with a research paper. There is a small but growing demand for lay summaries but the quality of lay summaries provided varies. To write a good summary requires a specific skill set. Researchers who are sometimes too close to their topic might not be the best people to write these summaries. Journal editors may also find it challenging to provide such summaries for peer-reviewed research articles.

With this in mind, BioMed Central conducted a short survey to assess the viability of offering paid-for professionally written lay editor summaries on journals and registration services. A SurveyMonkey survey was conducted in November 2012 (Additional file 1). Emails were sent to approximately 200 recent researchers who had used the ISRCTN registration service in Current Controlled Trials [5] and 370 BioMed Central journal editorial board members. There were almost 50 replies with the following findings:

●  Summaries are needed at ethics approval stage in 75% of cases.

●  Of the researchers surveyed, 79% do not involve lay people [enough] and may not even see why they should because lay summaries are ‘not difficult to write’.

●  Of the respondents, 79% either would not pay or did not know whether they would consider paying.

Current Controlled Trials aim to continue increasing the publication of lay summaries when their quality meets current standards. Current Controlled Trials will also follow changes in guidance as a result of implementations of the INVOLVE recommendations and will continue to discuss approaches for improving the volume and quality of lay summaries with relevant organisations, such as the other partners behind the UK Clinical Trials Gateway.

## Discussion

The authors welcome any comments, especially ideas on improving the transparency and accessibility for scientists and the public.

## Conclusions

All recent initiatives demonstrate that there are still a number of challenges in making current research both accessible and understandable by prospective participants. It is necessary to improve ‘signposting’, to direct the public to the information. Plain English summaries are seen as a good idea but very few people are willing to pay for improved content. Through extra guidance and funding, easier access and improved quality can be attained but this will take some time.

## Abbreviations

ISRCTN: International Standard Randomised Controlled Trial Number; NHS: National Health Service; NIHR: National Institute for Health Research.

## Competing interests

HF is an employee of BioMed Central, which publishes Current Controlled Trials. SD sits on the Advisory Group for Current Controlled Trials.

## Authors’ contributions

HF wrote the first draft of the manuscript. SD was involved in review and critical revision of the content before publication. Both authors read and approved the final manuscript.

## Supplementary Material

Additional file 1 Lay summary pilot survey. Click here for file
